# Children’s Comprehension of Irony: Studies on Polish-Speaking Preschoolers

**DOI:** 10.1007/s10936-019-09654-x

**Published:** 2019-07-16

**Authors:** Natalia Banasik-Jemielniak, Barbara Bokus

**Affiliations:** 1 The Maria Grzegorzewska University, ul. Szczęśliwicka 40, 02-353 Warsaw, Poland; 2 University of Warsaw, Warsaw, Poland

**Keywords:** Verbal irony, Non-literal language, Pragmatic competence, Social reasoning, Figurative language, Development of irony comprehension

## Abstract

We explored the topic of irony comprehension by preschoolers. Two hundred and thirty-one children (77 four-year-olds, 89 five-year-olds, and 65 six-year-olds) were tested with the Irony Comprehension Task (ICT, Banasik and Bokus, in: Poster presented at the psycholinguistics conference in Flanders, Berg en Dal, 2012). Participants were asked questions checking comprehension of the intended meaning behind an ironic comment. Four conditions were used for the ironic utterances: targeted (ironic comment was a reference to the addressee’s behavior), non-targeted (ironic comment was not a reference to the addressee’s behavior), with symmetric dyads (a child said the ironic comment to another child), and asymmetric dyads (an adult said the ironic comment to a child). All groups achieved high irony comprehension scores. The results show a significant difference in accuracy between the 4-year-olds and the 6-year-olds only. The youngest group more accurately understood ironic utterances that referred to the addressee’s action than those that did not, while older children did not show these differences. The aspect of who is speaking to whom was also significant only for the youngest children. These results provide important new insights into factors potentially influencing figurative language comprehension. Components such as participant structure and irony type require acknowledgement in the discussion on irony difficulty.

## Introduction

A significant share of everyday communication relies on the ability to understand non-literal language, for example irony (Capelli et al. 1990). There have been many attempts to define what constitutes an ironic utterance, though with limited agreement (Attardo 2000; Clark and Gerrig 1984; Kreuz and Glucksberg 1989; Kumon-Nakamura et al. 1995; Wilson and Sperber 1992). Some claim that defining irony is impossible (e.g., Nunberg 2001) and that it is more effective to characterize or describe irony rather than to try and create a definition (Barbe 1995). Nevertheless, several features are common for most definitions. These include: (1) the utterance representing two differing meanings, that is, the literal one and the intended, hidden one which might, but does not have to, be the inversion of the literal one (e.g., Anolli et al. 2002; Barbe 1995; Dynel 2014), (2) the speaker’s intention to use an indirect way of expression and their expectation for the recipient to comprehend the hidden meaning behind the ironic statement (Sperber and Wilson 1986), and (3) the knowledge shared between the speaker and the recipient, making it possible for the recipient to comprehend the intended meaning (Shugar et al. 2013).

Irony could also be referred to as sarcasm (cf. Lee and Katz 1998). Although these terms are often used interchangeably, some researchers point out that sarcasm is a form of verbal aggression (Haiman 1998; Martin 2007) or a form of irony (Muecke and Muecke 1969). Hence, we have decided to use the term *irony,* which might be considered more comprehensive.

Gibbs (2000) has shown that as much as 8% of all conversation turns are ironic. Yet irony is often misunderstood. Comprehension and production of verbal irony is a complex cognitive process (Ackerman 1983; Filippova and Astington 2008; Recchia et al. 2010; Winner and Leekam 1991), influenced by a range of individual differences (Ivanko et al. 2004). For these reasons, researchers agree that competence in irony understanding forms late in development (Pexman and Glenwright 2007). First, the addressee needs to understand that there is a duality of meaning in the utterance (the surface one and the real, hidden one). Then, the addressee has to refer to the knowledge shared with the addressee in order to arrive at their intended meaning. In other words, understanding irony means understanding the other person’s intention, grasping the two suggested meanings, and being able to choose the intended one. Because this mechanism is related to mental state attribution, figurative language comprehension is often linked to the development of the Theory of Mind (ToM, Banasik 2013; Huang et al. 2015).

Further understanding of irony comprehension by children and of the difficulty of its components is of interest to us for four reasons. First, the ability to grasp nonliteral meaning is an important part of communicative competence and hence, we believe it can shed more light on our knowledge of children’s language, social, and cognitive development (Raver 2003; Raver and Knitzer 2002; Zins et al. 2004). Second, by studying this emerging pragmatic competence, we focus on an element of a cultural phenomenon (Capelli et al. 1990; Gibbs 2000; Mitosek 2013). Irony is part of everyday conversations, media communications, and educational texts. It might even be argued that the ability to infer nonliteral meaning is a necessary skill in order for communication to be successful. Third, studies examining the age at which irony comprehension develops have yielded inconsistent results thus far (see Ackerman 1981; Dews et al. 1996; Filippova and Astington 2010; Harris and Pexman 2003; Pexman and Glenwright 2007; Sullivan et al. 1995). Finally, we hope to extend the investigation of this topic to children who speak languages other than English.

The main goal of the presented study was thus to explore irony comprehension by young, Polish-speaking children in three age groups (4-, 5-, and 6-year-olds). We were interested if such factors as the symmetry (i.e., two children speaking) or asymmetry (i.e., an adult speaking to a child) of the exchange or a reference to the addressee’s action or behavior in the ironic comment may play a role in the comprehension of irony.

The choice of the age group in this study was motivated by the previous literature on irony comprehension in children. Whereas the majority of studies show that children begin to understand ironic comments rather late in their development (Capelli et al. 1990; Glenwright and Pexman 2010 Filippova and Astington 2008; Winner 1988), there is some indication that this ability can actually be present much earlier (see Recchia et al. 2010). The current study was an attempt to further disambiguate these results.

## Irony Comprehension from a Developmental Perspective

Understanding irony is seen as a major milestone in the development of children’s social cognition (Peterson et al. 2012), as it requires understanding speakers’ beliefs, intentions, and attitudes (Filippova and Astington 2008).

Preschool children are capable of assessing the beliefs and intentions of other people correctly, though they have been found to predominantly fail to understand irony even until the age of nine (Demorest et al. 1983; Filippova and Astington 2008, 2010), with some studies reporting frequent failures by children as old as 13 (Demorest et al. 1983; Demorest et al. 1984).

Regarding irony in particular, researchers using various methodologies have come to the conclusion that 6-year-olds understand the incongruity between the surface and the intended meaning in a spoken ironic utterance (Ackerman 1983; Glenwright and Pexman 2010; Hancock et al. 2000; Harris and Pexman 2003; Sullivan et al. 1995; Winner and Leekam 1991), as well as the intentions of the speaker (Dews et al. 1996). They can also detect some pragmatic functions of irony (e.g., Andrews et al. 1986; Filippova and Astington 2010; Harris and Pexman 2003; Winner and Leekam 1991). Nevertheless, irony comprehension occurs late in development in comparison with other forms of figurative language: it is preceded by the ability to recognize similes and metaphors (Andrews et al. 1986). When looking at various types of irony, it has been shown that both sarcasm and hyperbole are easier for children to comprehend than understatement (Winner et al. 1987). Moreover, appreciation of critical ironic comments develops sooner than that of funny ironic comments (Dews et al. 1996) or of ironic compliments (Pexman and Glenwright 2007). Children who are not yet able to comprehend ironic comments usually misinterpret them by assuming them to be deception.

More recent studies claim that children as young as four are capable of comprehending the intended meaning of an ironic statement (Banasik 2013; Recchia et al. 2010) and that 3- and 4-year-olds show an emerging ability to recognize the communicative intent in simple utterances (Loukusa and Leinonen 2008).

Ackerman (1982) and Winner et al. (1987) found that memory was not a major factor in children’s inability to recognize ironic comments. However, despite abundant literature, we still do not fully understand what factors may facilitate irony comprehension.

Also, there has been little research on the understanding of ironic utterances in Polish-speaking children specifically (cf. Banasik 2013; Milanowicz and Bokus 2011). There has also been little research in general on cultural or regional differences in irony comprehension and usage. Most of the studies available describe results obtained within English-speaking communities.

### Irony as a Culture-Specific Phenomenon

According to the sociolinguistic theory of language variation, which claims that even speakers of the same language at some point develop differences in things like accent, vocabulary, or grammar (Labov 1972, 1996), it could be expected that the choice of certain linguistic forms might vary among communities, and hence it is important to study irony across locations and languages. Also, the way of communicating and directing utterances to others can be expected to be heavily culture-loaded (Park and Kim 2008). For the above two reasons, we thought it important to study verbal irony in a language which has not yet been analyzed in this respect.

Poland is thought to be a high-context culture according to Hall’s (1959) classification: It relies heavily on implicit communication and nonverbal cues. In such cultures, a message often cannot be understood without a significant amount of background information. Accordingly, in Polish culture, the use of indirect messages is a common characteristic of both interpersonal interaction and media communication (Lubecka 2000; Ogiermann 2009). What is more, the preference for indirectness in Polish culture is thought to be strengthened by the its history. During the periods of occupation and later the communist regime, artists and journalists looked for ways to avoid censorship. Irony emerged as a useful tool for social critique, revealing absurdities and hypocrisies (Kowalczyk 2017). For example, trying to sum up the climate of Polish literature from the 19th and 20th centuries, Głowiński (2002) claims that irony is a “constant element of human existence.” Żardecka-Nowak (2004), referring to philosophers such as Rorty, also writes of the “ironic attitude” which is characterized, among other things, by perceiving language as a tool for dealing with reality. Though no quantitative studies exist on this topic to the best of our knowledge, such a large body of literary criticism concerned with irony in Polish literature and culture points to a potentially significant factor.

The sociolinguistic theory of language variation claims that even speakers of the same language at some point develop differences in things like accent, vocabulary, or grammar (Labov 1972, 1996). Thus, it could be expected that the choice of certain linguistic forms might vary among communities, and hence it is important to study irony across locations and languages. Also, the way of communicating and directing utterances to others can be expected to be heavily culture-loaded (Park and Kim 2008). For these reasons, we thought it important to study verbal irony in a language which has not yet been analyzed in this respect.

### Types of Irony

There is a myriad of theoretical classifications of irony. It has been suggested that various types of irony have different levels of difficulty for children (Aguert et al. 2017; Bosco et al. 2013; Burnett 2015).

A previous Polish study (Banasik 2013) showed that young children can comprehend simple ironic utterances where irony is based on the reversal of meaning. Both 4- and 5-year-olds scored high on an irony task and there was no significant age-related improvement.

Other studies have shown that children understand ironic criticism earlier and more accurately than ironic praise (Pexman and Glenwright 2007). Utterances containing an element of reference to the negative outcomes of the addressee’s actions are also understood by English-speaking children sooner than ones without such information (Demorest et al. 1983).

For the purpose of this study, we focused on two types of ironic comments: (a) *targeted* irony that refers directly to the addressee in a critical fashion, describing the negative outcomes of their (i.e., the target’s) actions (for example, “Nice job!” when somebody spills juice on the speaker’s clothes), and (b) *nontargeted* irony that comments on reality or a certain situation without referencing the addressee (target; for example, “What great weather for a walk!” when it starts raining heavily).

For instance, a story containing targeted irony is as follows:

Staszek and Piotrek are playing in the backyard. There are puddles and there is mud on the ground. Staszek falls down. He stands up and his pants are wet and muddy. “You are so clean!” says Piotrek.

**Fig. 1:**
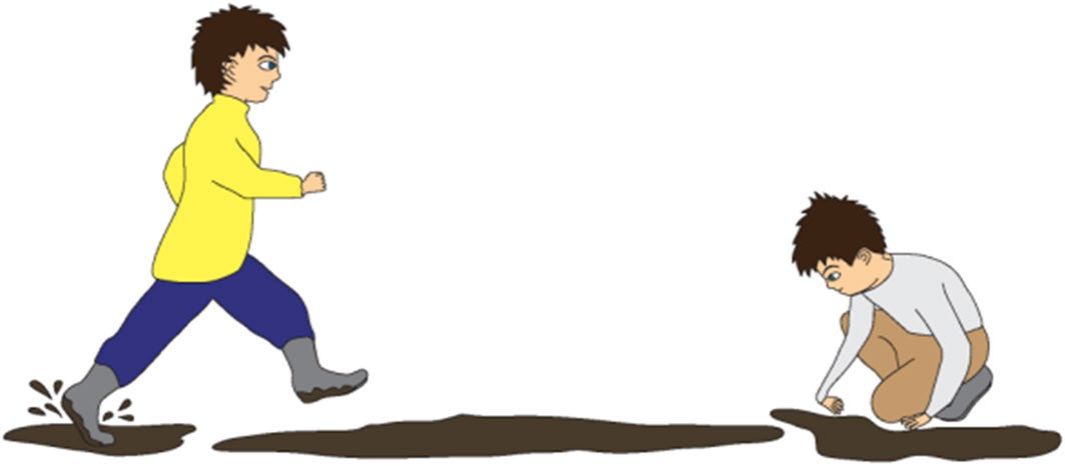
Illustration accompanying the beginning of one of the story with ironic comment (targeted irony)

**Fig. 2:**
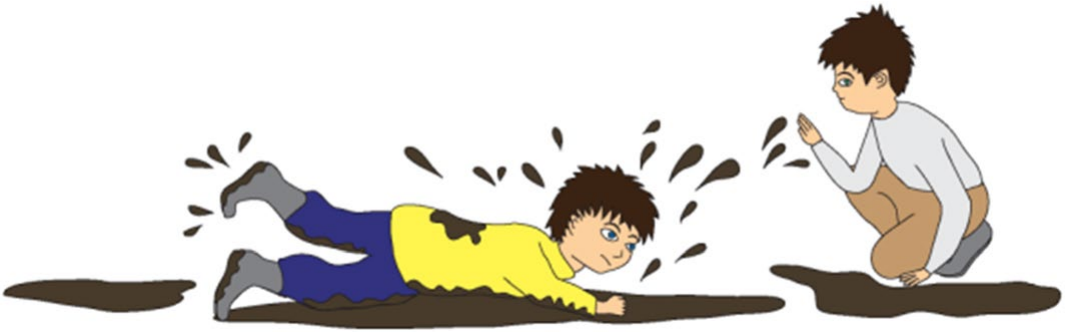
Illustration accompanying the climax of one of the story with ironic comment when the (targeted irony)

**Fig. 3:**
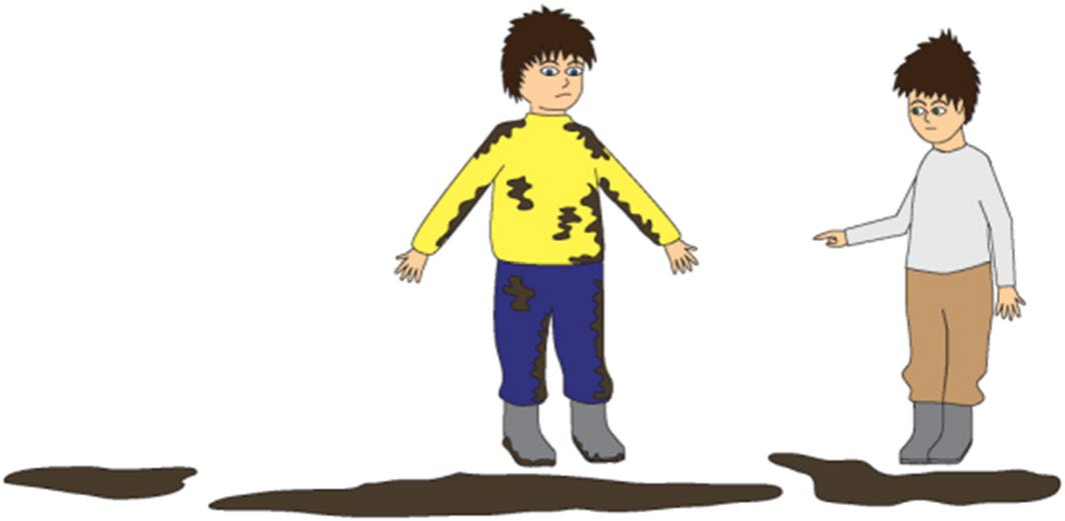
Illustration accompanying the moment of ironic utterance said by a character in one of the story with ironic comment when the (targeted irony)

Staszek is simultaneously the target and the addressee of the message. Hence, “You are so clean!” can be perceived as criticism or playful ridicule of Staszek’s clumsiness or lack of attention. Figures 1, 2, and 3 present the pictures that are shown to children who are given this task.

Below is one of the stories that include a nontargeted ironic comment:

Gosia thinks spinach is yucky. She never eats it. They are having spinach for lunch today. Gosia does not want to eat the spinach. She says to her friend, “Oh! My favorite food!”.

**Fig. 4:**
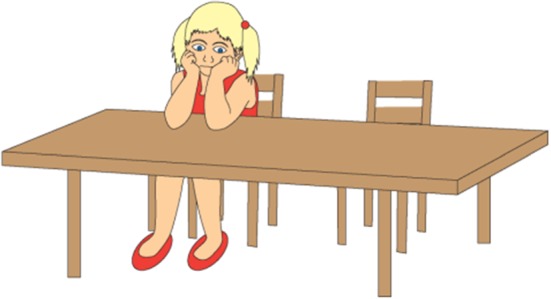
Illustration accompanying the beginning of one of the story with ironic comment (nontargeted irony)

**Fig. 5:**
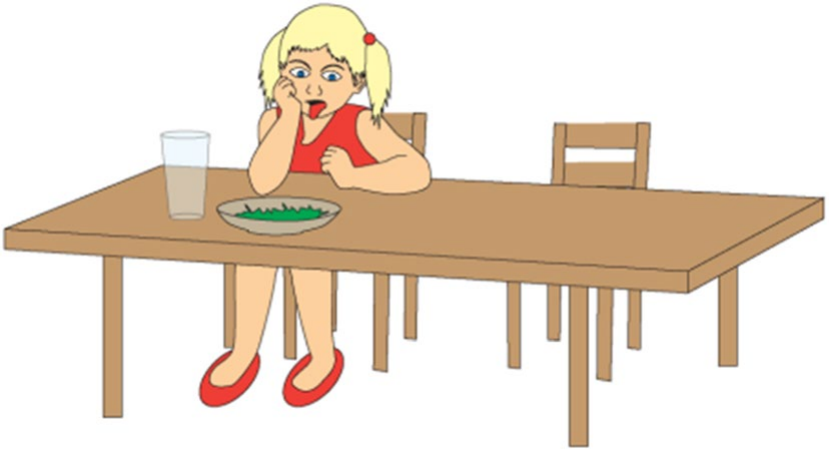
Illustration accompanying the climax of one of the story with ironic comment when the (nontargeted irony)

**Fig. 6:**
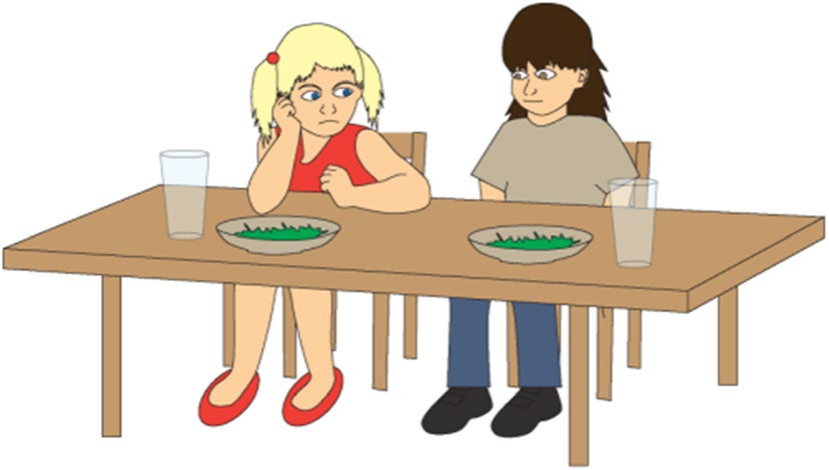
Illustration accompanying the moment of ironic utterance said by a character in one of the story with ironic comment when the (targeted irony)

The friend is not responsible for Gosia having spinach for lunch. Hence, the ironic comment does not refer to something that the addressee has done. Figures 4, 5, and 6 present the pictures that are shown to children who are given this task.

Using the Irony Comprehension Task (ICT, Banasik and Bokus 2012; Banasik 2013), a standardized irony comprehension task intended for children, narrows the scope of our study by focusing on the most simple and easy to comprehend type of irony. However, taking into account our emphasis on age and tracking the developmental trajectory of irony competence, the ICT allows for obtaining more precise results. As such, the current study presents just one stage of a wider research project.

### Participant Structure

*Participant structure* (Philips 1972) refers to the relations between the actors in an event, or, in other words, between the interlocutors in a conversation (see also: Bokus and Kałowski 2016).

Information on the relationship between the speakers was proved to be a useful cue for recognizing the speaker’s intention behind ironic utterances (Kreuz 1996; Kreuz et al. 1999; Kreuz and Link 2002; Slugoski and Turnbull 1988). Also, the type of relationship between the interlocutors affects the ratings of communicative goals related to irony, that is, comments spoken to solidary addressees were perceived as funnier, more teasing, and less status-changing than statements made to nonsolidary addressees (Pexman and Zvaigzne 2004). However, this was only the case for adults. It has been demonstrated that when processing ironic utterances, children do not use information on whether the speaker and the addressee are friends, strangers, or enemies (Pexman et al. 2005). However, in their research on Italian-speaking children, Massaro et al. (2013) suggested that irony comprehension may be dependent on *who is speaking to whom*. According to the results of their study, children tend to understand ironic utterances better if the interlocutor is an adult rather than another child. For example, if a mother, rather than a sibling, was the speaker, irony was understood more easily. The authors explain this by suggesting that adults introduce social norms and the principles of communication to children; hence, children recognize adult communicative competence better than that of other children. Moreover, it is probably most often adults—especially mothers (Recchia et al. 2010)—rather than other children (including siblings) who direct nonliteral language to children.

### Processing of Irony

Irony processing is explained mainly through two opposing hypotheses. The first one, described as the classic model of irony comprehension, is the *standard pragmatic model* (SPM; Attardo 2000; Booth 1974) and its revised version (Giora 1995, 1997, 2002, 2003; Giora and Fein 1999; Giora et al. 1998, 2007). The SPM claims that the literal meaning of the ironic utterance is processed first and the information incongruent with this meaning is rejected. Thus, an ironic utterance is always initially misunderstood. In the next stage, the intended (ironic) meaning is accessed. A number of studies confirm this model, showing that irony processing takes more time (i.e., is more difficult) than processing literal utterances (Dews and Winner 1999; Schwoebel et al. 2000).

A competing hypothesis is the *direct access model* (DAM, Gibbs 1994), which states that pragmatic knowledge is activated immediately when decoding the meaning of an ironic utterance. Figurative language is thus comprehended in the same way as literal language, that is, in a one-stage process. A number of empirical studies support this hypothesis as well (Gibbs 1986; Ivanko and Pexman 2003). The DAM is also the basis for Shelley’s (2001: 778) *bicoherence* theory. According to this theory, human cognition is organized to maximize conceptual coherence, that is, logical interconnection and consistency. One possible verification of this hypothesis involves comparing the reaction times in processing of figurative versus literal utterances. Pexman (2008) also proposed a constraint-satisfaction approach, which also claims that various cues are processed as soon as the ironic statement is uttered and that the ironic interpretation is arrived at once there is enough evidence to support it.

## The Present Study

### Research Questions and Hypotheses

As was described in “Irony Comprehension from a Developmental Perspective” Section, a large body of research has shown that 6-year-old children begin to comprehend irony, but it is unclear whether younger children do as well. Hence, we decided to start with the group that we would expect, based on previous results, to score high on a simple irony task and include two younger groups. The youngest group, based on various studies, can be expected to achieve lower scores on the same task.

The first, and to the best of our knowledge the only study where the Irony Comprehension Task (ICT) was used with monolingual Polish children (Banasik 2013), has introduced an interesting and much needed tool to study simple irony comprehension in an accessible form (i.e., where the language used is strictly controlled for difficulty). On the other hand, it presented results that were not compelling: there was no main effect of age, which might have been caused by the very modest sample size (46 children in three age groups). Hence, another study using the ICT is needed.

The aim of the present study was thus to investigate whether Polish-speaking children as young as four are capable of correctly interpreting ironic utterances. We also wanted to check for accuracy and reaction time differences in irony comprehension between 4-, 5- and 6-year-old Polish-speaking children. The reaction time needed to reply to comprehension questions about ironic and nonironic utterances (only correct answers were taken into consideration) was included as a correlate of processing speed and difficulty of the presented material. Examining potential differences between these age groups could yield important data on the developmental dynamics of irony comprehension.

Another aim of the study was to explore the impact of the type of irony used in the utterances (targeted vs. nontargeted irony). Additionally, we tried to examine the role of the participant structure: Does it matter who is speaking to whom? Do preschool children comprehend ironic utterances more accurately when speaking with an adult rather than a child?

The specific research questions were thus as follows:

1. Can Polish-speaking children as young as 4 years old understand simple ironic comments?
2. Are there significant differences in irony comprehension between 4-, 5- and 6-year-old Polish-speaking children?
3. Are there significant differences in irony comprehension between these age groups depending on whether targeted or nontargeted irony is used?
4. Are there significant differences in irony comprehension between these age groups depending on the participant structure (Who is speaking to whom, i.e., symmetrical vs. asymmetrical participant configurations)?

Reaction times might be an indicator of task difficulty (Keele 1986; Sternberg 1998). Thus, comparing reaction times in addition to accuracy of answers, we anticipated to be able to infer if the task was easier for older than for younger children. Also, if the reaction times to questions about the comprehension of ironic comments were significantly higher than for the literal comments, this would be an argument for the SPM.

Previous research on irony comprehension in adults used reading time. Some such studies found that participants do need more time to read ironic than literal statements (e.g., Dews and Winner 1999; Filik and Moxey 2010; Akimoto et al. 2012), while others—that ironic sentences might be processed as fast as literal ones (Gibbs 1986; Ivanko and Pexman 2003). Using the visual word paradigm with children, no effect of sentence type in reaction time was found (i.e., no support for the SPM, Climie and Pexman 2008; Nicholson et al. 2013).

We expected children as young as four to have above-chance results in the ICT (Hypothesis 1). We expected differences between 4-, 5- and 6-year-old children speaking Polish (Hypothesis 2), with older children scoring higher and answering faster. That is, that 6-year-olds would be the fastest and most accurate whereas 4-year-olds would be the slowest and have the lowest accuracy. We also predicted that the intended meaning in targeted irony would be easier to recognize than in nontargeted irony (Hypothesis 3), given previous findings that children understand ironic criticism more easily than ironic praise (Gibbs 1986; Matthews et al. 2006). As for participant structure, on the basis of Massaro et al. (2013), we assumed that accuracy will be higher and reaction times shorter in child–adult dyads than in child–child dyads (Hypothesis 4).

### Methods and Participants

Two hundred and thirty-one monolingual, Polish-speaking preschool children were presented with the ICT (Banasik and Bokus 2012). Then, they were asked to answer a series of close-ended questions. Offering two response choices, they checked the children’s understanding of the utterances’ intended (i.e., nonliteral) meaning. Additional data was collected by asking those children who have given correct responses an open-ended question about the speaker’s intentions behind the utterance.

The analysis was conducted for three age groups: four- (*n* = 77, age in months: *M* = 48.0; *SD* = 2.9), five- (*n* = 89, age in months: *M* = 60.0; *SD* = 3), and 6-year-olds (*n* = 65, *M* = 71 months, *SD* = 4.1).

### Materials

The ICT (Banasik and Bokus 2012) is a story comprehension task consisting of 12 stories. Six stories include one character addressing another with a counterfactual utterance interpreted as ironic by adult speakers of Polish. The other six, used as control items, involve utterances interpreted as a literal comment on the situation depicted. The stories were controlled for length (number of words in each story), morphosyntactic complexity (simple or compound, but not complex, sentences were used), difficulty of words (words already acquired), character dyads (child to child vs. adult to child). Within the test trials, half of the ironic comments in the stories were spoken by an adult to a child and the other half were spoken by a child to a child. This allowed us to analyze the possible influence of (a)symmetry of the interlocutors on irony comprehension. Similarly, three of the stories included a targeted ironic comment and three**—**a nontargeted one.

The conditions were not evenly crossed in the design, that is, within the six ironic stories, there were three symmetrical and three non-symmetrical dyads. Also, there were three targeted and three non-targeted utterances. However, within the three symmetrical dyads, two of the ironic utterances were targeted and one was non-targeted, and within the three non-targeted utterances, two included non-symmetrical dyads and one a symmetrical one. It was important to make the task relatively short and since we did not have a hypothesis about interaction of these factors, we decided not to add more stories in order to make it possible to fully cross the conditions.

Children were presented with the ICT on a computer with a touchscreen and then asked to choose one of the two given meanings of the ironic statement, the literal and the nonliteral (intended). When the question was auditorily played to the child, there were two pictures on the touchscreen, showing the character in the literal and in the non-literal situation, For example, when the comment was “You are so clean!” a picture with the character who had clean clothes on and one with the same character in dirty clothes were presented.

While the nonironic comments were read in a neutral tone of voice, the ironic ones were marked by distinct prosodic features of hyperarticulation: lengthened phrases, increased fundamental frequency, and increased signal energy. This was motivated by earlier research showing that speakers across languages tend to use these prosodic modulations in natural speech when using irony (e.g., Bryant 2010; Cheang and Pell 2008; Rockwell 2000). The speaker’s relationship with the addressee was fully counterbalanced across ironic and literal stories. In half of the stories, the characters were children only, while in the other half, there was an adult and a child character. In the latter case, the adult was the one uttering the ironic statement. The relationship information was presented to the participants both through pictures and verbally: It was said that the mom, uncle, or father is speaking to the child in the case of the asymmetrical dyads, and a friend or a sibling in the case of the symmetrical dyads.

Using a touchscreen for the experiment made it possible to measure reaction times. The indicator of the accurate answer was the child choosing the correct answer to a question about the meaning of the story by pressing the touchscreen where the corresponding picture, but not the distractor, was displayed.

**Fig. 7:**
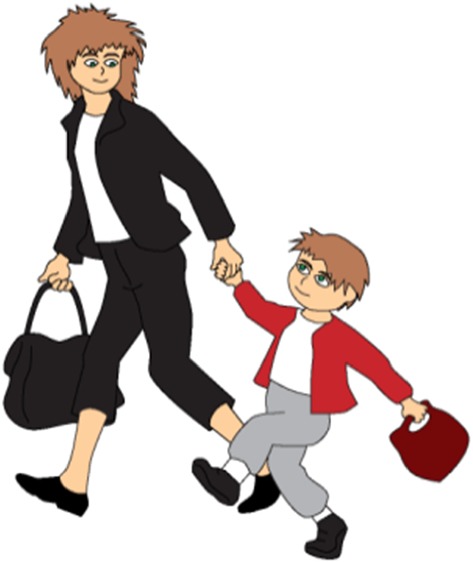
Illustration accompanying the beginning of one of the story with ironic comment (nontargeted irony, asymmetric dyad)

**Fig. 8:**
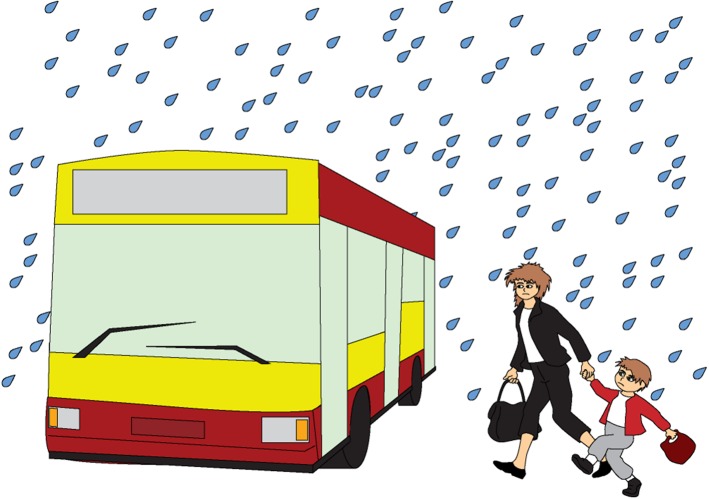
Illustration accompanying the climax of one of the story with ironic comment when the (nontargeted irony, asymmetric dyad)

**Fig. 9:**
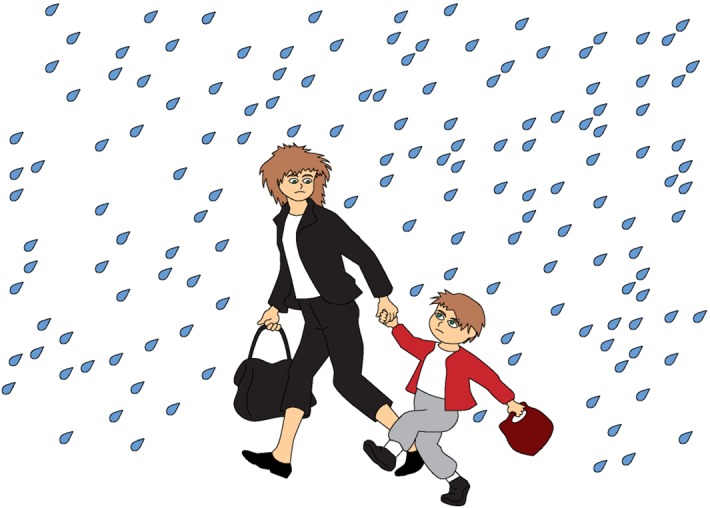
Illustration accompanying the moment of ironic utterance said by a character in one of the story with ironic comment when the (targeted irony, asymmetric dyad)

Figures 7, 8, and 9 show examples of the visual stimuli used. The captions represent the auditory stimuli that accompanied the pictures.

Krzyś is coming back from preschool with his mom. They want to get back home soon.

It starts raining. Krzyś and his mom are running to catch their bus. But the bus door closes and the bus leaves without them.

‘We are so lucky today!’ says Krzyś’s mom.

The total ICT score was calculated by summing up the correct responses to the comprehension questions (maximum score: 12).

Additionally, each story in the ICT ended not only with a closed-ended question checking the children’s comprehension of the character’s utterance, but also with an open-ended question: “Why do you think X said that?” The responses to open-ended questions were then analyzed within the framework of content analysis (Hsieh and Shannon 2005).

### Procedure

The study was conducted on preschool premises. Children were tested individually. The experimenter first tried to establish rapport with the child and then acquainted him or her with the equipment (computer, touch screen, sound recording device) and the procedure. The instruction included information about how to respond to the questions that will be asked “by the computer” and some brief training where the child was asked “Where is X?” and then had to respond by touching a picture on the screen that corresponded to the verbal label used. The questions were literal and the training consisted of six trials. After that, the test proper proceeded. The stories in the ICT were prerecorded and played back to the children during the session, together with the picture stimuli on a large (21.5 in.) touchscreen. After a trial test, the children responded to the questions by touching the screen and answering the questions aloud, which was recorded by the sound recording device. While the accuracy question was played, the two pictures (showing the intended meaning of the utterance and the distractor) were displayed on the screen.

The time was measured using E-prime software for each story separately, the measurement beginning with the start of the story, from which we later extracted the time between the start of the question being read out until the moment when the child picked the response on the touchscreen. The time was controlled with precision of up to a millisecond. The child was verbally praised for every response. No corrective feedback was given.

## Results

### Differences Between Age Groups in Irony Comprehension

We used SPSS to analyze the data for the three age groups (4-, 5- and 6-year-olds). As our data was characterized by distributions that diverge from the normal, nonparametric tests were used. The Kruskal–Wallis tests for independent samples were conducted in order to compare the groups’ ICT results (total score, max. = 12).

The analysis showed significant differences in ICT scores between the age groups (χ_2_ = 9.69; *p* < .05). The post hoc Mann–Whitney *U* test (taking into account the Bonferroni correction based on multiplying the significance by the number of tests performed, in the case of the presented tests: 3), presented in Table 1, showed significant differences between 4- and 6-year-olds, *Z* = − 3.009, *p* = .009, but not between 4- and 5-year-olds or 5- and 6-year-olds. Six-year-olds had significantly higher average results than 4-year-olds, while 4-year-olds did not differ significantly in understanding ironic statements from 5-year-olds. Five-year-olds performed as well as their older counterparts.

**Table 1:** Post-hoc tests: differences between age groups in the correct indication of the meaning of an ironic statement after taking Bonferroni’s correction into account

Multiple comparisons: Mann–Whitney U test						
Bonferroni test						
Dependent variable	Age	N	Avg rank	Sum of ranks	Asymptotic significance (two-sided)	Z
Correct answers in the interpretation of ironic statements	4	77	77.8	5935.00	0.23	− 1.761
	5	89	89.06	7926.00		
	5	89	73.06	6502.00	0.26	− 1.692
	6	65	83.58	5433.00		
	4	77	63.06	4855.55	0.009	− 3.009**
	6	65	81.50	5297.50		

**The difference in means is significant at 0.01

These results concern the questions about ironic comments. There were no statistically significant differences between the age groups regarding the frequency of correct answers to questions about nonironic comments: 4-, 5- and 6-year-old children did equally well in interpreting the meaning of a simple literal statement.

**Fig. 10:**
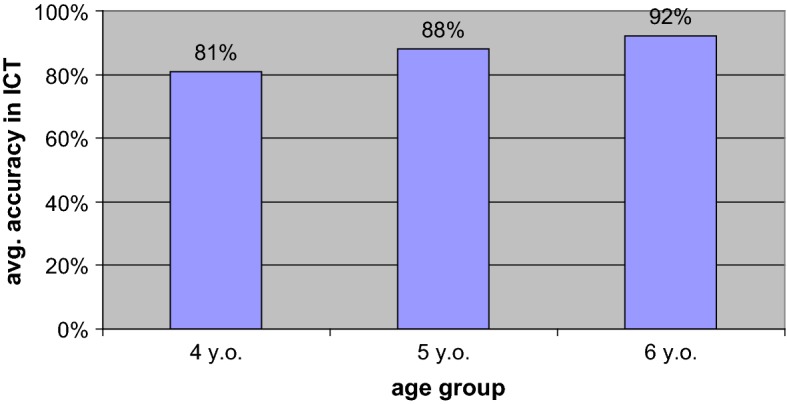
Accuracy in the Irony Comprehension Task (ICT). Understanding of intended meanings in ironic utterances, average results, age

The children’s ICT results, that is, the correctness of answers to questions about ironic comments, are shown in Fig. 10.

### Comprehension of Simple Ironic Utterances by 4-Year-Old Children

The results indicate that 4-year-olds scored high on the ICT (average accuracy of 81%). This means they were able to correctly recognize the proper meaning of an ironic utterance, provided they were not hindered by its grammatical or lexical complexity.

### Targeted Versus Non-targeted Ironic Utterances

Significant differences between in targeted and nontargeted ironic utterance comprehension was found only in the group of 4-year-olds (*Z* = − 2.46, *p* = .01). Targeted (i.e., expressing criticism of the addressee) statements were understood more accurately. No significant differences were noted in the older groups.

### Symmetric Versus Asymmetric Dyads

Significant differences in understanding ironic statements within symmetrical (child–child) versus asymmetrical (adult–child) dyads was found only in the group of 4-year-olds, *Z* = − 2.50, *p* = 0.01. They understood ironic utterances in asymmetric dyads more accurately, while 5- and 6-year-olds could recognize the intended meaning in the ironic utterances equally well in both conditions.

### Differences in Understanding Various Types of Irony Between Age Groups

No significant differences in understanding irony within symmetrical dyads were found between the age groups. However, there were age group differences in understanding ironic utterances in asymmetrical dyads. Six-year-olds understood such statements significantly better than 5-year-olds did, and 5-year-olds—better than 4-year-olds.

Nontargeted irony was best understood in the oldest age group, and the least in the youngest. There were no significant differences between the age groups in understanding targeted ironic statements.

### Reaction Times

The Kruskall-Wallis test, a nonparametric equivalent of the ANOVA variance test, was used to compare children 4-, 5- and 6-year olds years in terms of mean reaction times of the correct responses. Analyses did not reveal significant differences in reaction times of children providing correct answers to questions about ironic statements (χ_2_(2) = 0,626; *p* = 0,731).

The Wilcoxon test showed that children needed more time when they were responding to questions about the ironic statements than when they replied to the questions about the literal ones (Z = − 9.04, *p* < 0.001). We only compared reaction times of giving a reply to the questions for which correct answers were given.

### Responses to Open-Ended Questions

In case of stories where children have shown they understood the intent of the character speaking ironically, that is, gave the correct answer to the first closed-ended question, the answer to the following open-ended question (“Why do you think X said that?”) was also analyzed.

The responses to the open-ended questions were analyzed qualitatively within the content analysis framework, which involves classifying the responses into categories distinguished on the basis of patterns of meaning (Hsieh and Shannon 2005). Based on the data gathered, the following categories of responses were distinguished:

1. No answer, negative answers (e.g., “I don’t know”), inconclusive answers, or answers referring only to the superficial meaning, such as:
  [“Well, my favorite food!” when the character does not like spinach].
  “Because… I don’t think I know”
  “Because she likes it” (referring to the superficial meaning)
  “Well because it’s her favorite food (referring to the superficial meaning)
  “Because she didn’t try it” (inconclusive)
  “You answer” (turning to the experimenter, inconclusive answer)
  “Just because” (inconclusive)
2. Answers referring to the speaker’s intended meaning:
  “Because she didn’t like spinach”
  “Because in fact she didn’t like this food”
  “Because she doesn’t like it”
3. Answers indicating the duality of meaning by (a) a reference to the character’s internal state (intent or emotion) or by (b) metalinguistic means, including references to the statement’s function:
  [“Well done!” said to a boy who just spilled juice on a clean tablecloth]
  “Well, because he was angry that Krzyś spilled the juice on the tablecloth” (referring to the character’s mental state)
  “Because it was sarcasm,” “Because it’s a saying” (metalinguistic explanation)
  “He was joking” (referring to the statement’s function)
  [“How lucky we are today!” said when the characters have just missed the bus]
  “Because a mom sometimes says things the other way round and it’s called a metaphor“, “You say that when you’re not lucky at all, the door slams shut in your face and you don’t have anywhere to go” (metalinguistic explanation)
  “To cheer him [the son] up” (referring to the character’s intent)
  “Because she thought they would make it to the bus, but it wasn’t true” (referring to the character’s mental state)

Inconclusive answers and answers indicating the utterance’s literal/superficial meaning (Category 1) in the total response set decreased for 5-year-olds (31% of all responses) compared to 4-year-olds (42%, test of proportion for independent samples *z *= 3.12, *p *< .001). For 6-year-olds, it remained at a similar level (26%) as for 5-year-olds (no statistically significant difference was found).

Indicating the intended meaning of an ironic utterance (Category 2) was the most frequent category of answers for all the age groups. Its proportion increased in 5-year-olds (67%) compared to 4-year-olds (56% *z *= 3.20, *p *< .001) and remained at a similar level (63%) in 6-year-olds. No statistically significant difference was found between five- and 6-year-olds for this category of answer. Finally, indicating the duality of meaning by referring to a character’s internal state or by metalinguistic explanations (Category 3) also increased with age. For 4- and 5-year-old children, it accounted for just 2% of all explanations, growing to 11% for 6-year-olds (*z *= 5.63, *p *< .001).

Replying to subsequent questions in the ICT, many children gave answers belonging to different explanation categories. Among 4-year-olds, only 4% justified the character’s use of irony (Category 3) at least once. Among 5-year-olds, 3% of children gave such a justification at least once, and among 6-year-olds, this was as much as 38%. Table 2 presents the percentages of children in the different age groups that gave at least one explanation belonging to a given category. Each child answered six questions; thus, they could potentially refer either multiple times to each category or to just one or two of them.

**Table 2:** The of children whose explanations could be classified into the given category at least once

Age group	N	% Children whose explanation was classified to category 1	% Children whose explanation was classified to category 2	% Children whose explanation was classified to category 3
4	76	92	98	4
5	53	67	100	3
6	65	78	98	38

Proportion of children who gave at least one answer from a given category in their explanation of an ironic utterance.

It is worth underlining that almost all children**—**regardless of age**—**provided at least one explanation showing they understood the speaker’s intended meaning and gave it as the motivation for the speaker to use irony. There was a perceptible developmental change between 5 and 6 years of age, as almost 40% of the 6-year-olds (and only 3% of 5-year-olds) referred at least once in their explanation to justifications for using irony (Category 3; *z *= 5.30, *p *< .001).

## Discussion of Results

The main finding of our study is that Polish-speaking children can understand linguistically simple ironic utterances as early as at the age of four.

This result adds to the discussion about the development of irony comprehension. Although a similar result has already been obtained in a natural-conditions observation study on Canadian children (Recchia et al. 2010), many researchers postulate that as a competence developing late in the linguistic and social development of a child, irony understanding occurs a few years later. Additionally, as was discussed above, results on the emergence of irony understanding obtained from one language/culture might have limited generalizability to others. Thus, aside from a replication, the current results also constitute new evidence regarding the Polish language. Additionally, the research presented here used a method similar to those used to show irony understanding at the age of 6, 7, or 8 years. However, a very significant modification was introduced: We strictly controlled the lexical and grammatical complexity of the stimuli used. Thanks to the use of early acquired words and simple structures familiar to the youngest children, the results obtained with the ICT should not indicate a false lack of understanding of intentions that could result from language difficulties.

Laval and Bert-Erboul (2005), who studied comprehension of sarcastic requests in French-speaking children aged 3-6, while interpreting the results of their study that indicated that the youngest participants did not understand not only sarcastic, but also literal requests, noted that the experimental material which is based on just auditory information, but not visual one, may not be suited for children this young.

In our study, children were presented both auditory and visual information, which might have made the task more age-relevant.

The results showed differences in the level of irony understanding depending on the children’s age. They are, however, slightly different than could have been expected. The data show that differences in accuracy are only relevant between 4- and 6-year-olds, but not between 4- and 5-year-olds or 5- and 6-year-olds. This is a very interesting result, suggesting that, perhaps, the processes essential for understanding simple irony are progressive in their development, without any abrupt increases in skills. As we have assumed, the differences concerned only the irony understanding, while the meaning of the literal statement was understood at a similar, high level of accuracy in all age groups. Interestingly, irony comprehension in asymmetrical dyads and comprehension of nontargeted ironic utterances differentiated children across the age groups. Four-year-olds better understood targeted than non-targeted irony, but no such difference was noted in the older age groups. We did expect that targeted irony would be easier to understand. Prior research has shown that ironic criticism is the simplest form of irony and the one understood the earliest by children. Thus, it is surprising that this was not the case for 5- and 6-year olds in our study. It seems that employing the least complex type of irony in our study has increased comprehension accuracy for the age group that has the greatest difficulty in understanding irony in general. In other words, criticism could be an additional cue for the children who need it to recognize the intended meaning. Older children, on the other hand, might understand irony on a higher overall level, such that an additional cue no longer affects their results. Another possible explanation is that non-targeted irony (an utterance not related to the interlocutor) was referencing a situation that was contrary to the expectations and hence included not only verbal irony, but also situational irony, which could strengthen the message’s meaning and make it easier for older children to recognize the speaker’s intention. Although it is not clear why this information would be a guide for older children, but not for younger ones, there has been a study that indicated a difference between younger and older children in being able to use contextual cues to interpret sarcastic utterances. Laval’s and Bert-Erboul (2005) found that while 5-year old children score high in the interpretation of sarcastic requests as long as they have intonation cue, they evolve towards context-based comprehension at the age of 7. In other words, younger children need to hear the sarcastic intonation to interpret the utterance correctly and they fail to comprehend it in context condition, whereas older children pass the task in both the context and the intonation condition.

Another explanation is related to the quite high results for ironic statements in general. With a high level of performance in the ICT, facilitation does not play a role, since the average results were still high. In other words, arriving at the correct meaning behind the ironic statements in the ICT was easy enough for the children so that the more favorable conditions (i.e., containing more cues) did not influence their results. A similar situation was observed with symmetrical versus asymmetrical condition.

There were no significant differences in the understanding of irony in symmetrical and asymmetrical dyads in 5- and 6-year-olds, nor in the entire sample. Only the youngest children scored higher on asymmetrical rather than symmetrical dyads. Again, a possible explanation that we find most appealing is that features facilitating irony understanding, that is, additional cues that make the process easier only have influence when the task is relatively difficult. When understanding certain statements is not a major challenge for children, the results are high regardless of any additional facilitation.

The reaction times of correct answers to questions about ironic statements also did not differ between the age groups. This result is quite surprising, but can be explained by the difficulty of translating the response times into actual processing times for such young children. It is conceivable that the time the children needed to respond was determined not by their cognitive readiness to answer the question, but rather by temperamental features, excitement related to curiosity, and the desire to play with the touchscreen, or, on the contrary, shyness (causing long reaction times).

Reaction time was included as an additional variable that could provide us with information on how processing spoken utterances changes with age, but in light of the obtained results, it can be considered an inconclusive variable.

Interestingly, however, the difference between the reaction times for answering questions about ironic and literal utterances were significant. The questions about ironic utterances yielded significantly longer response times than those about literal utterances, which is consistent with the modular theory or SPM.

The qualitative analysis of the children’s replies to the open-ended questions in the ICT distinguished three categories. The first one did not give any motives for speaking ironically, while the other two suggested the correct interpretation of the speaker’s intent. In the second category, this was done by indicating the actual, intended meaning of an ironic utterance (different from the literal, superficial meaning), and in the third**—**by indicating the actual meaning together with an additional reference to the character’s internal state or by explaining the use of irony through metalinguistic elements.

The older the children were, the better they were able to explain someone’s use of a given utterance, pointing more often to the ironic duality of meaning. It is possible this is connected both with a better understanding of ironic utterances themselves, that is, greater language competence, and with a better developed ToM, enabling the children to account for people’s beliefs and communicative intent (Banasik 2013; Bokus and Hernik 2015; Gałkowska et al. 2010). Intention as a ToM component takes part in the causal (mentalist) explanation of behaviors and often figures in their verbal description, justification, evaluation, and prediction (Bokus and Hernik 2015, p. 31).

## General Implications, Limitations of the Study and Future Directions

Children from all age groups performed quite well on the ICT. This is a valuable contribution to a significant body of research which has so far suggested that 5- and even 6-year-olds are at very early stages of comprehending that people do not always mean what they say, and so they have difficulties in comprehending ironic utterances (e.g., Hancock et al. 2000). Our results suggest that this capacity is already developing at the age of four. The high scores of 4-year-olds in our study also allow us to assume that the development of figurative language comprehension skills may begin even earlier than we have found, which might potentially be noticeable in experimental conditions if it were possible to further remove the linguistic and cognitive load of the task. The children’s results in the ICT also lead to the question of the source of this competence. The issue of children’s exposure to ironic language likewise remains an unexplored topic. A potentially fruitful avenue of research could involve observational studies similar to the one by Recchia et al. (2010). It would also begin to examine how possible intercultural differences influence the development of irony comprehension, or whether children in different cultures/languages are exposed to different forms and types of irony.

It is important to acknowledge that we used a type of irony that is considered easiest to comprehend**—**the kind where the meanings of the literal and figurative statements are opposite to each other (for instance, saying “Great weather for a walk!” to mean “The weather is bad for a walk”). Thus, the dissonance might be spotted without as much effort or additional cues as would be needed for other types of ironic expressions. However, this should not be used as an argument to diminish the impact of the results. The ICT was specifically constructed with regard to young children’s linguistic skills. The words used in the stories were ones that were acquired relatively early and the grammar was simple. With this, we hoped to measure the comprehension of ironic statements not hindered by complex vocabulary and grammatical constructions. This might not always have been the case in previous research. Thanks to this, we believe that we, in fact, studied the comprehension of ironic utterances and not the level of vocabulary or grammar development. Though the contents of the ICT have limited our examination only to simple, reversal-based irony, they have allowed us to examine it in greater detail, yielding more accurate and reliable results. It can be expected that as the complexity of the ironic stimuli employed increases, so does the number of variables that need to be controlled. By carefully delineating the scope of our study and using a validated task, we have thus managed to obtain significant and clear-cut results. It is important to note the development of ironic language comprehension, even if it does not yet allow for reliable recognition of the real meaning behind more complex and subtle forms of figurative language. The finding that 4-year-olds are able to decode the meaning behind some forms of irony is relatively new, regardless of the ironic forms used.

However, one limitation of the current study is that we have not controlled for instances of ironic utterances that are familiar (i.e., conventional) versus unfamiliar. It might be expected that familiar ironic comments would be processed differently than novel ones. A more thorough, detailed study is needed to investigate this issue. Another limitation was that targets and dyads were not perfectly crossed in the study design. Although we did not form an interaction hypothesis, this should be included in future studies. Nevertheless, the fact that the study was conducted with non-English speaking children may be very important and not only replicate previous results, but also extend their applicability. As was discussed in “Types of Irony” Section, Polish culture shows a preference for indirectness and figurative language, including irony. This may translate into exposure of children to ironic language at an earlier age. As a consequence, Polish children might be trained in decoding the correct meaning behind the ironic statement to a larger degree than some of their English-speaking peers. If this is the case, the high scores in an irony comprehension task for Polish, but not American or Canadian children, might well be justified by the use of figurative speech of their closest environment.

However, we need further studies that would look into this question, since our predictions about the role of culture are merely speculative. The results related to the form of irony that should be easiest for children proved to be very interesting. The facilitation effect that we expected was confirmed only in the youngest group, which may indicate that some cues that are useful for recognizing irony at a certain developmental stage cease to have an impact along with the further development of this ability. This could be an interesting idea for further research examining the developmental dynamics related to the understanding of nonliteral statements. Further investigations and replications with different groups, tasks, and languages are needed to explain and verify these results.

Further studies should also focus on investigating children’s comprehension of the pragmatic functions of verbal irony. It would be interesting to explore what children think of the person who delivers an ironic comment. It has been stated in the literature that it is the function of criticizing rather than the humor function that appears earlier in a child’s development. Examining differences between various languages and/or cultures in this regard could also yield potentially significant results. In the future, a new irony comprehension task should also be constructed in order to explore other types of irony. Among the indications for future research, we would like to mention further analyses of our results, as well as further studies on similar topics.
